# Walking kinematics in young children with limb loss using early versus traditional prosthetic knee prescription protocols

**DOI:** 10.1371/journal.pone.0231401

**Published:** 2020-04-10

**Authors:** Mark D. Geil, Zahra Safaeepour, Brian Giavedoni, Colleen P. Coulter

**Affiliations:** 1 Department of Exercise Science and Sport Management, Kennesaw State University, Kennesaw, Georgia, United States of America; 2 Department of Human Performance and Health, University of South Carolina Upstate, Spartanburg, South Carolina, United States of America; 3 Division of Prosthetics and Orthotics, Department of Orthopedics, Children’s Healthcare of Atlanta, Atlanta, Georgia, United States of America; Holland Bloorview Kids Rehabilitation Hospital, CANADA

## Abstract

The traditional treatment protocol for young children with congenital or acquired amputations at or proximal to the knee prescribes a prosthesis without a working knee joint, based in part on the assumption that a child learning to walk cannot properly utilize a passively flexing prosthetic knee component. An alternative to this Traditional Knee (TK) protocol is an “Early Knee” (EK) protocol, which prescribes an articulating prosthetic knee in the child’s first prosthesis, during development of crawling and transitioning into and out of upright positions. To date, no study has compared samples of children with limb loss at or proximal to the knee using TK and EK protocols. The purpose of this multi-site study was to examine kinematic outcomes during walking in separate groups of young children in an EK and a TK prosthesis protocol, along with a population of children without lower limb amputations. Eighteen children aged 12 months to five years were recruited for this study at two clinical sites, six in each of the three groups. Children in the two prosthesis groups had unilateral limb loss and had been treated either at one site with the TK protocol or at another with the EK protocol. Children in the EK group achieved swing phase prosthetic knee flexion averaging 59.8±8.4 degrees. Children wearing prosthetic limbs walked slower than age-matched peers. In most instances, walking speed and step length increased with age in the EK group, similar to the control group. However, this trend was not observed in the TK group. Clearance adaptations were present in both limb loss groups. Observed adaptations were twice as prevalent in the TK group versus the EK group; however, the groups differed in age and etiology. Children with limb loss provided with an articulating knee component in their first prosthesis incorporated knee flexion during swing phase and showed fewer gait adaptations than children in the TK protocol.

## Introduction

Limb loss creates unique mobility challenges in child development. Congenital limb deficiency is the most prevalent cause of childhood limb loss, with a rate of 3.5 to 7.1 per 10,000 births, followed by acquired amputation due to trauma and disease [1, 2].

Pediatric prosthetic rehabilitation is made more difficult for the clinician due to the changing functional mobility demands of the child and critical developmental milestones. Two important factors are the timing at which a child with limb loss is introduced to their first prosthesis, and the appropriate selection of prosthetic components [3]. During organization of a child’s musculoskeletal, vestibular, visual, and proprioceptive systems, the ability to maintain balance and upright posture gradually develops [4]. This organization is not independent from the child’s prosthetic limb. Ideally, the prosthesis should allow the infant to progress through typical developmental milestones such as sitting, squatting, kneeling, crawling, and independence in other physical activities. When transitioning from crawling to walking, an ideal prosthesis would provide mobile knee flexion necessary for crawling and stable knee extension needed for standing and walking. For clinicians, consideration of the developmental importance of kneeling, squatting, and crawling must be balanced with the stability required to transition to walking.

There is controversy regarding timing of prosthetic knee provision in children. For example, the most recent *Atlas of Amputations and Limb Deficiencies* provides conflicting advice. Scott-Wyard writes, “For a child with above-knee limb deficiency, the first prosthesis should not include a mechanical knee unit because of the need for increased function and stability [5].” In a later chapter, Coulter writes that infants with a knee in their first prosthesis “have demonstrated the ability to control a prosthetic knee and incorporate knee functions in early developmental activities [6].”

The conventional treatment protocol for young children with limb loss at or proximal to the knee prescribes a prosthesis without a flexing knee joint to provide a stable base for the development of standing and walking [2, 7, 8]. Smith and Burgess state, “The knee unit should be eliminated or locked in extension until the child is ambulating well and demonstrates proficient use of the prosthesis [7].” Historically, this “Traditional Knee” (TK) protocol has been utilized for two main reasons. First, past prosthetic knee joints were too large for infants and toddlers [2, 9]. Second, a general assumption was made that, as walking develops, stability is preferred over mobility. The first reason is obsolete, since appropriate pediatric knee components now exist [10, 11]. The second assumption is still widely held, since control of a passive prosthetic knee requires selective firing of residual limb muscles at appropriate phases of the gait cycle, acting both concentrically and eccentrically. However, to our knowledge, there is no published evidence that supports these assumptions, and lack of available knee flexion can hinder both swing phase clearance and the acquisition of age-appropriate functional activities.

Alternatively, an “Early Knee” (EK) protocol prescribes an articulating knee joint in the child’s first prosthesis, usually when the child achieves pull-to-stand around 12 to 18 months of age. The knee is a free-swinging joint with no active extension assistance or stance control [12–15]. Previous research has shown biomechanical advantages of the EK protocol. Geil et al. showed that infants with limb loss using a prosthetic knee locked in extension crawled with slower velocity and reduced cadence. However, a more typical “step-through” crawling pattern was seen in the unlocked knee condition, with less asymmetry [13, 14]. Geil and Coulter showed that clearance adaptations were reduced in the EK protocol, and that children achieved an average of 70.4° swing phase knee flexion in the articulated prosthesis side [12]. It is noteworthy that these studies did not include children in a traditional protocol. Instead, they used children in an EK protocol as their own controls by locking their existing prosthetic knee joint into full extension. Wilk et al. examined gait in children who were initially in a TK protocol, after training and transitioning to an unlocked knee, and finally after one year with the unlocked knee. They found that one year of walking with an articulating knee resulted in less asymmetry and reduced circumduction [15].

A gap that remains in the literature is an examination of gait in developing children in both TK and EK protocols compared to a control group. Therefore, the purpose of this study was to measure spatiotemporal and kinematic gait parameters for young children in each knee prescription protocol and age-matched typically developing children without amputations.

## Materials and methods

This study was a multi-site comparison with a convenience sample of 18 children aged 12 months to five years (Table 1) in three groups: six with unilateral lower limb loss at or proximal to the knee at a site where the EK protocol is the standard of care, six with unilateral lower limb loss at or proximal to the knee at a site where the TK protocol (with no prosthetic knee) is standard, and six age-matched typically developing controls without amputations (C). Children with limb loss used their prosthesis daily without assistive devices and had the same knee protocol from the time of their first prosthesis.

**Table 1:** Subject demographics. PFFD = proximal femoral focal deficiency.

	Age (m)	Sex	Mass (kg)	Height (cm)	Amputation		Foot	Knee	Socket, suspension
					Side	Presentation			
C1	15	M	10.5	75.6					
C2	47	F	21.0	112.0					
C3	67	F	19.2	104.0					
C4	68	F	22.5	117.0					
C5	32	F	12.5	91.0					
C6	21	F	11.5	81.0					
EK1	26	F	7.9	80.0	R	PFFD	TRS Little Foot	3R38	Ischial weight bearing (PFFD extension), TES belt
EK2	69	M	24.0	123.0	R	PFFD	College Park Truper	Total Knee Junior (1100)	Pelite liner (segmented socket with anatomical suspension)
EK3	48	F	15.5	101.0	R	PFFD	College Park Truper	3R38	KD (partial ischial weight bearing, partial distal end bearing), lateral fenestration straps
EK4	62	F	16.0	108.0	R	PFFD	Trulife Child’s Play	3R38	Ischial weight bearing (PFFD extension), TES belt
EK5	18	M	12.4	83.0	L	PFFD	TRS Little Foot	3R38	Ischial weight bearing (PFFD extension), TES belt
TK1	22	F	12.6	83.7	L	Fibular hemimelia	SACH		Quad, waist belt
TK2	38	M	17.6	106.0	L	Amniotic Band Syndrome	College Park Truper		Suction socket, sleeve
TK3	18	M	9.5	74.3	R	Fibular hemimelia	SACH		Quad, belt
TK4	31	M	14.5	91.4	R	Hypoplastic hemipelvis with absence of femur	SACH		Hip disarticulation (including Otto Bock Pedi hip joint)
TK5	17	M	10.4	78.5	L	PFFD	SACH		PFFD socket, lanyard suspension

The EK and C groups were assessed at Children’s Healthcare of Atlanta and Georgia State University. The TK group was assessed at Shriner’s Hospitals for Children in Shreveport, Louisiana. The fitting and alignment of each prosthesis was performed by an ABC board-certified prosthetist with pediatric experience at each hospital according to each site’s normal standard of care. The protocol was approved by human subjects review boards at each institution, and informed parental consent was obtained.

To ensure inter-lab reliability, a single typically developing four-year-old test subject was assessed at both sites within two weeks in a separate, approved protocol. Both laboratories utilized similar motion capture systems (Vicon T10 and T40 cameras at 100 Hz, Oxford Metrics, UK) with identical models and software (Vicon Nexus 2.0, Plug-In-Gait), and examiners followed standardized video training for marker placement [16]. Sixteen markers were attached according to the Plug-In-Gait lower body marker set, with bilateral markers on anterior and posterior iliac spines, lateral femoral condyles, lateral malleoli, second metatarsal heads, and posterior calcanei. Markers were also placed on the lateral femoral and tibial segments to define the frontal plane. The subject walked barefoot at a self-selected walking speed along a 10 m walkway with no support or external assistive devices. Ten trials were recorded. Comparison of results showed satisfactory consistency between labs. Small differences in placement of frontal plane markers on the thigh and shank segments caused tri-planar differences in kinematics, particularly in pelvis motion [17]. These placements were subsequently standardized for the main project.

Data collection for the main study followed the same protocol as the preliminary reliability study. For children with prosthetic limbs, prosthetic knee and ankle widths were measured directly and included in subject parameters. Spatiotemporal and kinematic parameters including walking speed, cadence, step length, knee flexion, pelvic obliquity, thigh abduction, and heel height were calculated and averaged across at least ten trials for each subject.

Three of the authors reviewed reconstructed 3D motion stick figures from each trial for each subject to provide subjective documentation of clearance adaptations. Observers were blinded to condition. The two clinicians (CC and BG) were primary observers, and the third (MG) broke any ties. These results were compared to numerical results for each, calculated as follows.

Hip hiking was quantified by measuring both the affected side frontal plane pelvic angle in mid-swing and the contralateral side hip ab/adduction angle [18]. We created a combined measure based on both of Kerrigan et al.’s measures [18], taken from mid-swing on the prosthetic side:

$$HH=\frac{\left((Prosthetic\;side\;pelvic\;obliquity)-(Contralateral\;side\;hip\;ab/adduction\;angle)\right)}{2}$$ (1)

A positive value means more pronounced hip hiking.

Pelvic obliquity was the maximum swing phase upward prosthetic side angle formed between the line connecting the two ASIS markers and a horizontal plane:

$$Pelvic\;Obliquity=\left(\frac{180}{\pi }\right).tan^{-1}\frac{\left(Z_{contralateral}-Z_{prosthetic}\right)}{\sqrt{{\left(X_{contralateral}-X_{prosthetic}\right)}^{2}+{\left(Y_{contralateral}-Y_{prosthetic}\right)}^{2}}}$$ (2)

where *x*, *y*, and *z* represent ASIS markers in the line of progression, medial-lateral and vertical axes, respectively [19].

Affected side thigh abduction in swing has been suggested as a measure of circumduction [18], calculated here as the angle between the lines formed by the thigh and knee centers and the vertical axis of the laboratory:

$$Thigh\;Abduction=\left(\frac{180}{\pi }\right).tan^{-1}\frac{Y_{knee}-Y_{thigh}}{Z_{knee}-Z_{thigh}}$$ (3)

where *y* and *z* represent knee and hip joint centers in the medial-lateral and vertical axes, respectively.

Vaulting was quantified by the maximum vertical displacement of the contralateral heel marker in midstance.

Descriptive statistics, including means and standard deviations, were calculated and compared between groups and between aged-matched pairs when possible.

## Results and discussion

Two children (one EK and one TK) were excluded from the final analysis. One was the only subject to use crawling as the primary mode of ambulation, and one had recently transitioned from a locked to an articulating knee at the time of the study. Therefore, the data from sixteen children were analyzed.

All EK and TK subjects had congenital limb deficiencies. The TK group had a mean age of 25.20 ± 9.04 months, and the reasons for amputation were varied (Table 1). The EK group had a mean age of 44.60 ± 22.15 months, and all had Proximal Femoral Focal Deficiency (PFFD). Differences between groups affected analysis. While age was similar at the young end (18 m vs. 17 m in the two prosthesis groups), ages in the EK group spread much older. An age-matched C was recruited for all TK and EK children, but direct age-matched comparisons between EK and TK were limited. Therefore, analysis focused on description of gait in each group, with consideration of the relative levels of development.

**Fig 1:**
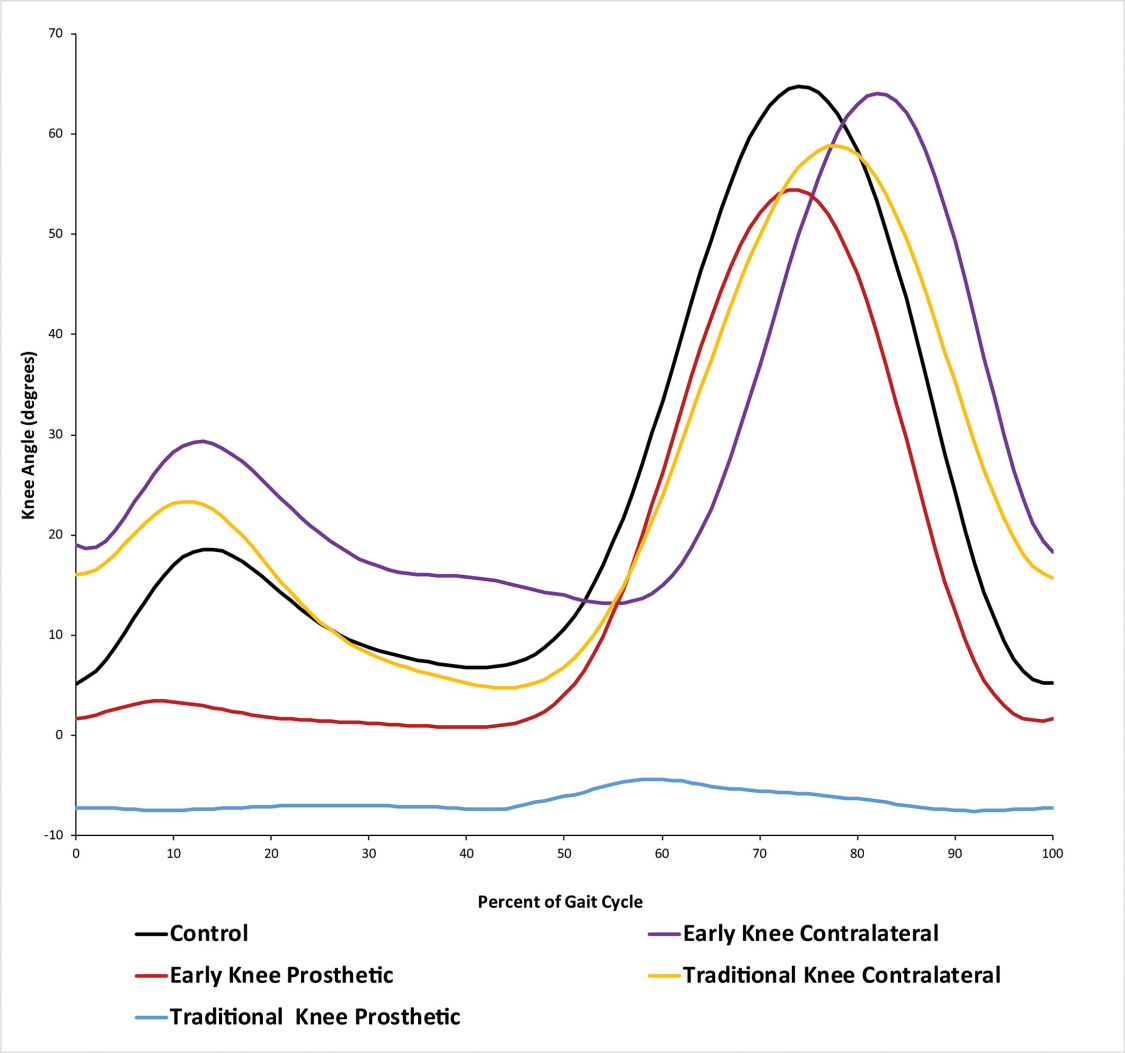
Knee flexion angles. Knee flexion angle (degrees), averaged for right and left for C, and displayed separately for prosthesis side and contralateral side for EK and TK.

All children in the EK group flexed their prosthetic knees during swing (Fig 1). Mean prosthetic peak knee flexion over all steps and all EK subjects was 59.8° ± 8.4°. The mean for the C group was 68.4° ± 3.8°. Contralateral side knee flexion was 69.3° ± 5.8° and 75.0° ± 5.5° for EK and TK, respectively.

**Fig 2:**
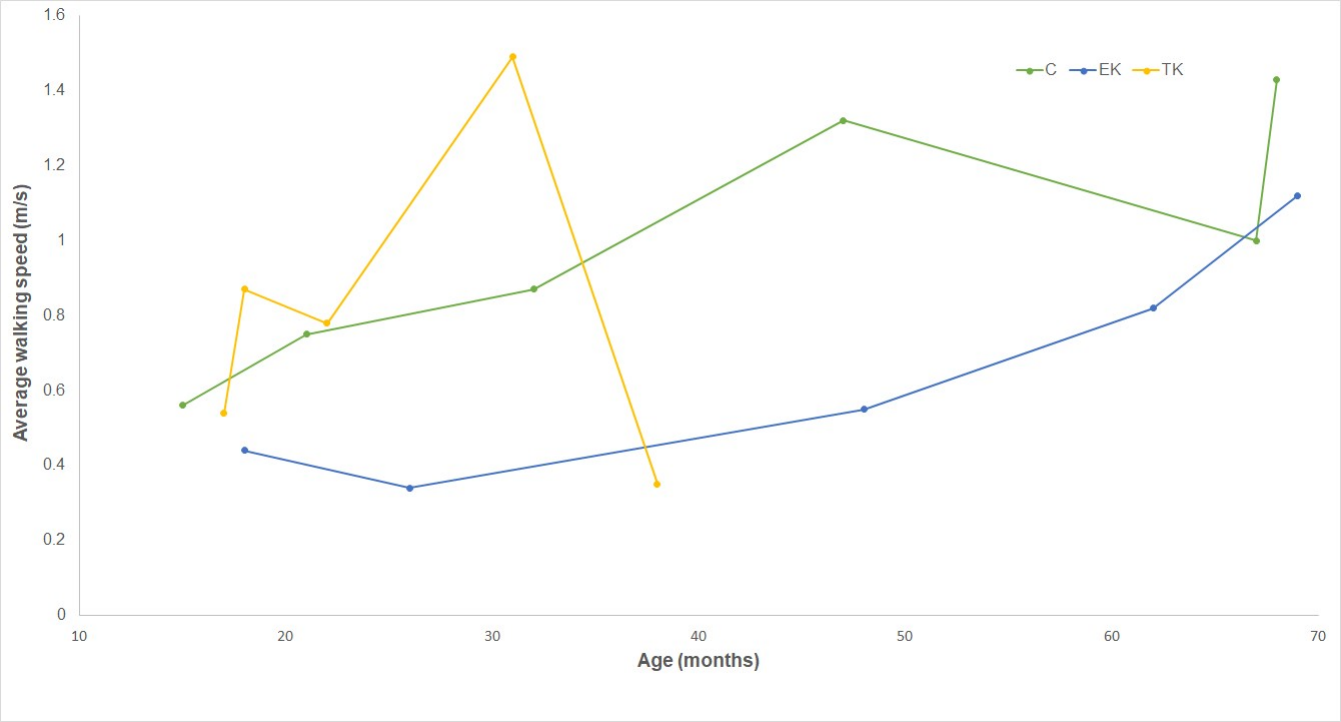
Relationship between walking speed and age. Average walking speed (m/s) for each subject in each group sorted by age (months).

Walking speeds were highly variable (Table 2). Two TK subjects showed clinically meaningful higher speeds compared to age-matched peers in the C group. These speeds were gained by an increase in cadence in both subjects, and by an increase in prosthesis-side step length in one. In general, children in the C and EK groups walked faster and with longer steps with increases in age. However, a similar pattern was not seen in the TK group (Fig 2).

**Table 2:** Spatiotemporal results in age-matched groups.

	Walking Speed (m/S)			Cadence (steps/min)			Prosthetic Step Length (cm)			Contralateral Step Length (cm)		
Age (m)	EK	TK	C	EK	TK	C	EK	TK	C	EK	TK	C
15–18	0.44	0.54	0.56	130	150	159	22.7	22.2	25.7	22.3	23.9	22.8
		0.87			189			33.6			26.0	
21–26	0.34	0.78	0.75	171	116	186		30.2	28.5		29.0	25.8
31–38		1.49	0.87		197	164		14.9	36.1		23.5	35.4
		0.35			121			55.6			46.1	
47–48	0.55		1.32	118		165	21.4		58.1	36.4		54.9
62–69	0.82		1.00	116		139	49.6		48.6	44.4		49.9
	1.12		1.43	158		176	53.4		57.0	47.3		56.0

Clearance adaptations (Table 3)—circumduction, hip hiking, and vaulting—were present in at least some subjects in both prosthesis groups, and some children exhibited multiple adaptations. Based on clinical observation, no adaptations were present in the C group, six were observed in the EK group, and twelve in the TK group. Measured adaptation results generally agreed with observational results, with exceptions particularly in the youngest subjects. For example, the youngest C subject showed the highest measure associated with circumduction, but this was due to a large step width common in toddlers and was not clinically observed as circumduction.

**Table 3:** Clearance adaptations. Numerical results for each adaptation (see Methods), along with shading to indicate observational results, with Red indicating the presence of the adaptation and Green indicating that it was not observed. Subjects in each group are sorted by age, youngest to oldest.

Subject	Circum-duction (degrees)	Hip Hiking (degrees)	Vaulting (mm)
C1	-33.5	17	3.5
C6	-3.8	2.9	5.5
C5	-7.2	1.3	10.1
C2	-3.7	3	9.2
C3	0.8	-1.8	12.7
C4	-3.1	-0.4	11.6
EK5	-19.3	18.5	9.4
EK1	-30.3	25.1	8.8
EK3	-30.2	8.6	31.2
EK4	-5.8	8.8	51.5
EK2	-14.4	14.5	15.3
TK5	-22.3	13	13.3
TK3	-20.1	18.1	5.2
TK1	-17.1	13.3	7.9
TK4	-18	17.9	46.3
TK2	-22.1	6.9	39.6

The TK protocol described herein has been the standard of care taught in orthopedic texts for decades in the United States, but the advice has promulgated without evidence. For example, Craig et al. in *Pediatrics* suggest a solid tube in the initial prosthesis and the addition of an articulated knee sometime between 37 and 72 months, but cite no evidence [20]. However, based on recent published evidence and anecdotal observation, some texts are now modifying this recommendation. Bowen and Otsuka in *Lovell and Winter’s Pediatric Orthopaedics* described the traditional protocol, but then noted, “In the experience at the authors’ center, as well as others, introduction of a prosthetic knee without a locking feature can be used as the first prosthesis when the child first pulls to stand [8].”

The most fundamental evidence necessary to inform pediatric prosthetic knee prescription is whether or not children can safely utilize an articulating prosthetic knee if one is provided to them. The five EK subjects all achieved swing phase knee flexion, followed by full extension for stable transition to stance phase. Individual means for peak knee flexion angle were variable and appeared to depend on factors including residual limb length and walking speed. Nonetheless, the overall mean of 59.8° was directly between values reported in Wilk et al [15], 49° and Geil et al. [12], 70.4°.

Variability in gait patterns in all groups was a complicating factor in interpretation of results. Even when standard deviations were low within a group, means between subjects were often quite different. In particular, the PFFD etiology in the EK group affected the results and was a limitation of the study. PFFD involves a variety of prosthetic fitting strategies, including distally located knee joints to allow for swing phase clearance and sitting [21].

It was interesting to note that some EK children still exhibited passive prosthetic knee flexion despite very distal placement of the knee joint center. For these children (EK5 in particular), the knee joint was so distal that swing phase flexion afforded very small gains in clearance. Geometrically, given a vertical thigh segment, placement of the knee from a vertically centered position (50% leg length) to a position 25% more distal (75% leg length from proximal) results in a clearance reduction of $\frac{L}{2}(1-\cos\theta )$, where *L* is the leg length and *θ* is the knee flexion angle. Distal knee placement can result in several centimeters of clearance loss. In addition, the small segment carries less mass and is therefore less apt to swing inertially. Nonetheless, these subjects still showed a reasonable swing phase knee flexion curve. This is promising, since the bilateral knee height discrepancy will diminish with growth while the child continues to learn necessary synergistic hip muscle action.

It is also important to note that children in the EK group maintained stance stability while utilizing the knee in swing, at least within the controlled laboratory environment. It is beyond the scope of this project to assess stability or instances of knee buckling during activities of daily living; however, anecdotal feedback from clinicians and parents in the EK protocol indicated no stability concerns beyond the falls typical of children learning to walk.

Lack of knee flexion necessitates a clearance adaptation. There was no single preferred adaptation in the TK group, and all TK children exhibited more than one adaptation. Four of five circumducted, all five showed hip hiking, and three of five vaulted. In the EK group, adaptations were present in most children, though they were fewer. However, as mentioned, several children in the EK group were older than those in the TK group.

For vaulting, a measure of around 35 mm was an approximate numerical cutoff between *No* and *Yes* observations, though TK5 was an exception. For hip hiking, a measure of about 7–17 degrees showed some *Yes* and some *No* observations. For circumduction, a cutoff of -20 degrees separated most *Yes* and *No* observations, except for TK1 and C1. These results differed from those for adults in Kerrigan et al. [18], who found an average of 9.8° thigh abduction in adults with stroke who had stiff-legged gait, and 3.9° in able bodied adults. This underscores the differences in our younger population, and the need for more refined numerical measures of clearance adaptations and severity in children.

Provision of a prosthetic component should also consider costs versus benefits. In this case, according to typical practice, the early provision of an articulating prosthetic knee joint carries no cost. Usually, the same knee would be provided anyway, just at a later point, and the service life of the knee is not affected by the early provision. In addition, in the United States, the code for reimbursement used to fabricate any prosthesis for a child with unilateral limb loss at or above the knee already includes provision for a knee joint. Code L5200 includes a “single axis constant friction knee [22].” The only available code without a knee joint is for a so-called “stubby” prosthesis, which is not appropriate for unilateral transfemoral amputations.

There were several limitations in this study. Foremost, the convenience sample resulted in small sample sizes and important difference in ages between the children in each limb loss group. This limited statistical analysis to description of each group and limited age-matched comparisons. Despite this limitation, consistent findings with respect to knee flexion enable conclusions to be drawn based on the data. In addition, this was a cross-sectional study of very young children. A longitudinal study would permit closer examination of the persistence of clearance adaptations following transition to a knee in the TK protocol.

## Conclusions

In conclusion, this study found that young children with limb loss will utilize prosthetic knee flexion and extension if it is available to them. Absence of an articulated knee, as in the TK group, requires the child to incorporate clearance adaptations in gait. Some children in the EK group exhibited no clearance adaptations. Given that there is often no monetary cost associated with provision of a knee joint in the first prosthesis, and the possibility of improved gait, the Early Knee protocol should be considered as a standard of care, replacing the traditional protocol.
